# The cognitive paradox of AI in education: between enhancement and erosion

**DOI:** 10.3389/fpsyg.2025.1550621

**Published:** 2025-04-14

**Authors:** Binny Jose, Jaya Cherian, Alie Molly Verghis, Sony Mary Varghise, Mumthas S, Sibichan Joseph

**Affiliations:** ^1^Department of Health and Wellness, Marian College Kuttikkanam Autonomous, Kuttikkanam, India; ^2^Department of Social Science, Lincoln University College, Petaling Jaya, Selangor Darul Ehsan, Malaysia; ^3^Vimala College, Thrissur, Kerala, India; ^4^Peet Memorial Training College, Mavelikkara, India; ^5^Department of Business Administration, Marian College Kuttikkanam Autonomous, Kuttikkanam, India

**Keywords:** artificial intelligence in education, Cognitive Load Theory, AI and critical thinking, self-determination theory, AI-driven learning strategies

## Introduction

Artificial intelligence (AI) is rapidly transforming learning through unparalleled levels of personalization, efficiency, and scalability (Govea et al., 2023; Mahmoud and Sørensen, 2024). AI-based technologies such as adaptive learning systems and intelligent tutoring systems are revolutionizing traditional pedagogical paradigms as they individualize instruction to the needs of particular learners (Alawneh et al., 2024). While these advances have the potential to enhance learning, they also raise significant questions about their implications on the cognitive development of students, particularly in critical thinking, problem-solving, and recall. Among the challenges is determining whether AI is an enabler of deep learning or inadvertently induces cognitive dependency—a cognitive offloading effect (Grinschgl and Neubauer, 2022; Kim et al., 2024).

This paper explores AI integrated education using Cognitive Load Theory (Schnotz and Kürschner, 2007) and Bloom's Taxonomy (Shaikh et al., 2021), the study examines the influence of AI on learning processes and cognitive elements such as cognitive engagement, retention, and higher-order thinking. Optimizing learning pathways is a vast area of study about how AI gets to these long-term cognitive trade-offs and ethical challenges, much less so. As AI is increasingly integrated into learning systems around the world, there is a pressing need to comprehend how it may be utilized to enhance learning potential without jeopardizing fundamental cognitive capabilities. Here we will introduce an end-to-end framework that will allow to critically analyze in which ways, and to what extent, AI will be beneficial (or detrimental) to the learning process; ensuring that it will always be employed to maximize the best potential for learning without undermining our most basic cognitive function.

## Cognitive offloading and AI's impact on cognitive skills

Cognitive offloading refers to the utilization of external aids to achieve cognitive tasks (Risko and Gilbert, 2016). While AI-powered tools certainly make it easier, they also reduce the opportunity for active recall and problem-solving, which are essential components of cognitive development (Çela et al., 2024; Grinschgl and Neubauer, 2022).

## Empirical evidence on AI's cognitive impact

### Memory retention

A study by Bai, Liu, and Su examines AI-based tools like ChatGPT and their impact on memory retention. While AI enhances personalized learning, excessive reliance may reduce cognitive engagement and long-term retention (Bai et al., 2023). Similarly, Akgun and Toker studied 73 information science undergraduates at a Pennsylvania university. Participants were divided into two groups: one engaged in pretesting before using AI, while the control group used AI directly. Results showed that pretesting improved retention and engagement, but prolonged AI exposure led to memory decline (Akgun and Toker, 2024). These findings suggest AI enhances accessibility but may weaken retention if overused. To maximize benefits, AI should support rather than replace human-driven learning strategies.

### Problem-solving and critical thinking

The impact of AI on critical thinking and problem-solving is becoming a concern more and more. Emmanuel Philip Ododo studied AI perceived problems in student retention and critical thinking in 206 vocational education students in the Akwa Ibom State, Nigeria. The results showed that AI posed significant threats to the male students showing more concern than their female peers. While AI aids vocational education, it has the potential to reduce cognitive engagement because the students may accept passively the information provided by AI without critical scrutiny (Ododo et al., 2024). The study emphasizes the need for AI-powered learning platforms that encourage source verification and independent thinking, ensuring that the students learn critically rather than passively.

### Creativity

Another area of serious concern is the impact of AI on creativity. An experiment on undergraduate students who enrolled for a Creative Thinking and Problem-Solving class at a leading Southeastern United States university studied the impact that AI tools, including ChatGPT-3, had on the divergent thinking ability of the students. Conducting a mixed-methods design using the Alternative Uses Task (AUT), the researchers found that AI-supported students scored better on fluency, flexibility, and elaboration when thinking up ideas but also found that the use of AI had liabilities, including cognitive fixation and lower creative confidence as the students over-relied on AI suggestions (Habib et al., 2024). These findings underscore the need to use AI in learning environments in a manner that maximizes its strengths and minimizes its liabilities so that AI becomes an enabler, not a constraint, on creative potential.

## Theoretical frameworks and AI in education

### Mapping AI applications to theoretical constructs

To better understand the relationship between AI in education and key psychological and cognitive theories, the following table categorizes AI applications based on their alignment with Cognitive Load Theory, Bloom's Taxonomy, and Self-Determination Theory.

#### Cognitive Load Theory (CLT)

Cognitive Load Theory (Schnotz and Kürschner, 2007) provides valuable insights into the manner in which AI impacts learning. AI has the potential to maximize the extraneous load through the elimination of redundant work, allowing the student to focus on more important cognitive operations. However, over-reliance on AI has the potential to reduce the germane load, which is needed for deep learning and the development of higher-order thinking skills. For instance, even as adaptive AI has the potential to personalize learning experiences and make difficult concepts easier, it must be done in a way that promotes effortful engagement rather than passive reception of information.

#### Bloom's taxonomy

Bloom's Taxonomy (Shaikh et al., 2021) categorizes cognitive skills from simple recall to higher-order thinking. The role of AI varies at various levels. AI applications may enhance recall and synthesis of information (lower-order skills), but the risk that AI may stifle more judgmental and analytical skills (higher-order thinking) exists. If students overuse AI-provided answers, their skill to develop independent critical thinking skills may be impaired. Therefore, AI implementation must be made in a way that enhances higher-order thinking along with the strengths of AI to automate lower-order thinking skills.

#### Self-determination theory (SDT)

Self-Determination Theory (Ryan and Deci, 2000) points to three psychological needs—autonomy, competence, and relatedness—that are critical to motivation and learning. AI's role in the academic environment has to be in harmony with these principles. While AI has the potential to create competence through customized learning, excessive dependence on AI could compromise autonomy. AI-based learning also has to provide human interaction and guidance to prevent the erosion of relatedness, keeping the students socially and emotionally engaged in the learning process.

## Implementation roadmap for AI in blended learning

To make sure that AI enhances learning without replacing necessary thinking functions, a well-planned implementation roadmap has to be adopted. Firstly, educators must do a needs analysis to determine where AI can be integrated and align with learning goals for cognitive functions. Secondly, selection of AI has to be thoughtful, with selected tools facilitating engagement and not dependency. Thirdly, AI has to be integrated in the classroom in a balanced manner, with space for individualized guidance but with teacher-driven conversations. And finally, monitoring and feedback mechanisms need to be instituted to measure the impact on student learning and make necessary adjustments accordingly.

## Real-world applications of AI in learning

### AI-powered adaptive learning in STEM

AI tutoring has increased student performance on standardized tests. Stanford research found a 15% increase in scores for students using AI platforms compared to traditional instruction (Top 6 AI Tools Revolutionizing Math Tutoring Techniques, n.d.).^1^ However, concerns remain about the building of conceptual understanding. Research by the University of Pennsylvania with Turkish high school students found that those using ChatGPT to practice performed worse on exams when compared with those who did not use the technology. While AI-assisted students answered correctly 48% more problems, their score on a concept understanding test was 17% lower (Barshay, 2024). These findings show that AI enhances procedural skill but does not necessarily create deeper learning. For optimal benefit, AI-based tutoring must be integrated with pedagogy that encourages critical thinking and active engagement.

### AI and language learning

The application of AI-based software has been found to be immensely useful in language learning. At the University of Baghdad, research centered on the contribution provided by AI toward the teaching of the English language, with the focus being on its contribution toward the performances of the students. The findings indicated that AI-based software, including speech recognition, chatbots, and virtual tutors, contributed immensely toward the communicative competence and fluency of the students. Additionally, more than 80% of the respondents believed that AI would be a major contributor toward language teaching in the future. While AI provided individualized teaching and real-time feedback, the research also indicated the need for human interaction for enhanced concept formation and engagement. Some students believed that additional training would be necessary to ensure the maximum utilization of AI in language learning (Khalil, 2024). These findings reflect the reality that even as AI might be a value addition to language acquisition, it must be an addition to the traditional learning process and not a replacement for human teaching.

### Addressing AI pitfalls in education

While AI has much to offer the learning process, it also presents real threats that must be addressed in order to use it responsibly. Most significant among these threats is the danger of over-reliance on AI, which lowers the quality and extent of mental work as well as the quality of the ability to solve problems. If students over-rely on AI-based answers, they might bypass necessary mental operations like critical thinking and self-reasoning (Correia et al., 2024). In order to counter this threat, instructors must incorporate reflection points, where students must describe AI-given answers in their own language. Periodic AI-free solving sessions and class discussions with fellow classmates also ensure active mental engagement, making sure that AI becomes an add-on, and not a replacement, for self-reasoning.

Another vital challenge is algorithmic bias, where AI models trained on unbalanced datasets could perpetuate educational inequalities unwittingly. This becomes a major issue when AI-based assessments or grading software favor some populations based on unbalanced data. To counter this, designers must include transparency mechanisms in the creation of AI models and diversify the datasets through the addition of multilingual and multicultural inputs (Chinta et al., 2024). Furthermore, the implementation of fairness-aware AI algorithms has the potential to reduce bias, with the effect that AI-based educational software increases inclusiveness and does not perpetuate inequalities.

A third concern is the risk that students who are highly reliant on AI-based learning experiences may lose motivation (Neji et al., 2023). If the thinking is done by AI, the students may lose motivation and engagement as well as the intrinsic motivation to learn and solve problems independently. To counter this, AI should be designed to develop autonomy through interactive, self-directed challenges. Gamification modules, adaptive problem-solving, and decision-making and creative input-requiring AI simulations can ensure motivation with the promise that the students are active agents in their learning process. AI should be a cognitive enabler that encourages deeper engagement and not a passive repository of knowledge.

## Conclusion

The application of AI in learning has a paradoxical character because it has the capability to be both a cognitive amplifier and inhibitor. In order to be maximally beneficial, AI must be designed as an enabler, rather than a substitute, fostering intellectual autonomy, problem-solving, and deep learning. According to Cognitive Load Theory, AI must decrease cognitive overload but sustain active cognitive engagement. According to Bloom's Taxonomy, AI must aid higher-order thinking and not enhance recall alone. Additionally, according to Self-Determination Theory, AI must generate autonomy and competence and not dependency.

AI should complement and not replace human instruction, with students doing higher-order thinking as the efficiency and personalization strengths of AI are leveraged. This calls on teachers to implement AI in a way that enhances self-directed learning and metacognitive thinking. AI designers and policymakers must ensure equity, transparency, and explainability to prevent biases in algorithms and make AI accessible to all students on the same level playing field.

Future research should consider the long-term impact of AI on thinking development, specifically on the storage of memories, critical thinking, and motivation in students. As AI reshapes the learning process, it becomes imperative to create pedagogically informed AI integration strategies that balance the efficiency provided by technology with the thinking skills that are necessary for meaningful learning. Maintaining AI-based learning as a force for empowerment, rather than dependency, will be crucial in the development of the future of learning.

## Footnotes

^1^https://www.ideta.io/blog-posts-english/top-6-ai-tools-revolutionizing-math-tutoring-techniques (accessed March 19, 2025).
